# Student Volunteering as a Solution for Undergraduate Health Professions Education: Lessons From the COVID-19 Pandemic

**DOI:** 10.3389/fpubh.2020.633888

**Published:** 2021-01-26

**Authors:** Ewelina Chawłowska, Rafał Staszewski, Agnieszka Lipiak, Bogusz Giernaś, Monika Karasiewicz, Dominika Bazan, Maria Nowosadko, Mateusz Cofta, Jacek Wysocki

**Affiliations:** ^1^Laboratory of International Health, Department of Preventive Medicine, Poznan University of Medical Sciences, Poznań, Poland; ^2^Department of Hypertension, Angiology, and Internal Medicine, Poznan University of Medical Sciences, Poznań, Poland; ^3^Promotion and Careers Department, Poznan University of Medical Sciences, Poznań, Poland; ^4^School of Foreign Languages, Poznan University of Medical Sciences, Poznań, Poland; ^5^Department of Preventive Medicine, Poznan University of Medical Sciences, Poznań, Poland

**Keywords:** health professions education, undergraduate medical education, student volunteering, service-learning, community service, emergency response, COVID-19

## Abstract

In response to the COVID-19 pandemic, many medical universities worldwide, including the Poznan University of Medical Sciences, launched student volunteering projects (SVPs). We examined our student volunteers' perceptions on the conditions, safety, costs and benefits of their participation in the SVP. Using this information, we attempted to assess the viability of SVPs as a solution for health professions education during and after the pandemic. The main research tool was a questionnaire on students' perceptions of their participation in the SVP. As a complementary qualitative method, we used semi-structured interviews with the volunteers. Our respondents (*n* = 158) perceived conditions and safety generally positively: most reported having personal protective equipment (89.24%), technical support (88.61%), and induction training (79.11%). Only 38.61% said they had access to psychological support. In our view, benefits (e.g., an opportunity to make new contacts or receiving positive reactions from patients and staff) seemed to outweigh costs. 65.82% of the respondents agreed that they learnt new interesting things. A majority noticed the development of their soft skills (social 86.08%; organisational 78.48%; stress management 68.99%), while 40.51% – the development of their medical skills. The interviews pointed to additional benefits for students such as gaining insight of the healthcare system, and costs such as distress caused by some patient interactions. We conclude that student volunteering could become a viable solution for health professions education. To maximise its educational potential, volunteers' needs must be explored, psychological support ensured, and opportunities for mentoring and reflection provided. The organisational framework of a SVP should be culturally sensitive.

## Introduction

In many parts of the world, student volunteering (SV) as a form of community engagement is not only an important part of the mission of higher education, but also a curricular activity forming an important part of experiential learning (1). What distinguishes SV from other forms of student engagement such as service-learning or internships is the focus and intended beneficiary of the engagement. In volunteering, the focus is on the service provided and the beneficiary is the service recipient (e.g., the patient receiving care). In internships, the main focus is learning, and the primary beneficiary is the student who learns. Service-learning, in turn, attempts to strike a balance between the service and the learning components (2).

In Poland, as well as in most Central and Eastern European countries, SV is not an element of university curricula (3). Under the Polish law, volunteering is distinct from mandatory forms of experiential learning such as internships (4, 5). While Polish medical universities widely endorse various SV initiatives, they do not treat them as a teaching tool that could support student learning.

The situation changed with the outbreak of the coronavirus disease (COVID-19), declared a pandemic by the World Health Organization on 11 March 2020 (6). It presented itself not only as a global public health emergency, but also as a challenge to health professions education. Lockdowns, restrictions on gatherings, shortages of healthcare personnel, and withdrawal of students from clinical settings forced medical schools worldwide to come up with a number of educational innovations. One of them was using SV to support health systems overburdened by responding to COVID-19 (7, 8). It turned out to be a good solution to staff shortages during the crisis (9–22).

In Poland, the incidence of COVID-19 in mid March 2020 was still relatively low at 3.3 total cases per 1 million population (23), but the pandemic-related preparedness procedures contributed to healthcare workforce shortages. They had already been evident before: in 2017, Poland had 2.4 doctors (OECD mean 3.5) and 5.1. nurses (OECD mean 8.8) per 1,000 population (24). In 2019, 54% of Polish doctors were employed at more than one health facility (25). In 2020, faced with the challenges caused by the pandemic, the government obligated health professionals working with COVID-19 patients to do it at only one facility (26). On 12 March, medical universities were asked to invite students to take part in the pandemic response by supporting epidemiological institutions (27). The shutdown of in-person teaching imposed on the same day (28) banned students from practical learning.

In response to that difficult situation, the Poznan University of Medical Sciences (PUMS), in close collaboration with student organisations, launched a COVID-19 student volunteering project (SVP). The first beneficiary institutions were PUMS teaching hospitals, but soon other institutions joined, including city hospitals, Sanitary and Epidemiological Inspection offices, outpatient clinics, laboratories, pharmacies, as well as the local Chamber of Physicians, which coordinated various field activities. PUMS offered three incentives to attract student volunteers: [1] gaining a credit for a compulsory internship; [2] postponed and more flexible assessment of e-learning outcomes; [3] concessionary prices for PUMS accommodation. The project, widely promoted through official university channels and student-led campaigns, soon became very popular: 1,126 students (19.19% of all students) of 16 (out of 19) fields of study taught at PUMS took part between 12 March and 30 June. Consequently, the SVP replaced practical learning for about a fifth of PUMS students during the first wave of the pandemic.

Such a wide involvement might indicate that SV has a potential not only to support the health system during emergencies, but also, under some conditions, to become a solution for improved health professions education in post-pandemic times. It is then the role of medical universities to initiate and develop such SVPs which might complement and reinforce educational outcomes. In this article, we take a closer look at the Poznań SVP and similar projects to see what lessons towards achieving this aim they can offer to countries such as Poland – countries without traditions of incorporating SV in university curricula. As a starting point for these considerations, we use perspectives obtained from our student volunteers.

There are multiple theoretical approaches to volunteering (29, 30). We chose a cost-benefit approach as our theoretical framework. It sees volunteering as an activity undertaken by an individual when the perceived benefits of volunteering outweigh its costs (31, 32). Some of the benefits of volunteering quoted in literature include personal (e.g., learning, career opportunities, self-development), social (e.g., respect from others), and normative ones (e.g., fulfilling the civic duty, following the need to help others). Volunteering may also involve similar categories of costs: personal (e.g., time, effort, stress), social (e.g., negative social reaction), and normative ones (e.g., frustration from lack of progress towards intended goals) (31, 33). Thus, one of the aspects we explored in our study was volunteers' perceptions on selected costs and benefits of SVP participation.

The main objective of our study was to examine volunteers' perceptions on the conditions, safety, costs and benefits of their participation in the SVP. We wanted to find out which benefits incentivised our volunteers more: the internship credit or the normative benefits of volunteering. We also intended to check if volunteers noticed any educational benefits of participation. Using this information, we attempted to assess the viability of SV as a solution for health professions education during and after the current COVID-19 pandemic.

## Materials and Methods

Our main research tool was an anonymous web-based survey designed by means of Webankieta.pl (Get Feedback Racino, Sadowski, Skowronek s.j., Poland) and advertised by PUMS e-mail service, on intranet and social media. Survey completion was voluntary. Ethical review and approval was not required for the study in accordance with the local legislation and institutional requirements.

We used a self-developed questionnaire (see English translation in Supplementary File 1) with 35 questions of various types (open-ended, yes/no, multiple choice, 5-point Likert scale of attitude). There were 3 questions about students' demographic details, 3 about their previous volunteering experiences, and 29 about their COVID-19 voluntary service: volunteering placement and responsibilities, safety and conditions of the service (contact with COVID-19 patients, training, equipment, technical support, etc.), costs of volunteering (time spent, clash with educational responsibilities), benefits of volunteering (skill development, feeling needed, meeting new people, increased self-esteem, etc.), as well as the importance of helping others and receiving an internship credit as incentives to participate. In the final question we asked if volunteering confirmed the student's choice of the field of study.

The survey was available online from 4 May to 30 June 2020. After the survey closing date, we divided the study sample into groups according to the answers provided. The undecided answers to scaled questions were not included in the analyses. The groups were compared using the chi-squared test (Statistica, version 13, TIBCO Software Inc., USA) to determine significant differences between group pairs. *P* values below 0.05 were considered statistically significant.

To gain a wider view of the SVP, we used semi-structured telephoned interviews with four volunteers, including one representative of the Student Union. We asked open-ended questions about SVP benefits, costs, safety and organisation, reasons for participation, opinions on the internship credit as an incentive, and opinions on making SV a curricular activity in the future (see English translation of the topic areas in Supplementary File 2). We also asked follow-up questions whenever a topic needed elaboration. This allowed us to obtain more detailed information on those aspects which had been explored by means of closed-ended questions in the survey.

## Results

Until 30 June 2020, 161 student volunteers submitted the questionnaire. Three responses were incomplete. The remaining 158 responses were included in data analysis. The characteristics of the respondents and details of their volunteering service are presented in Table 1.

**Table 1:** Characteristics of the survey respondents and details of their volunteering service (*n* = 158).

Age (in years)	Mean	23.3
	Median	23
Sex	Female	72.78% (*n* = 115)
	Male	27.22% (*n* = 43)
Field of study	Medicine	70.89% (*n* = 112)
	Nursing	6.33% (*n* = 10)
	Medical laboratory science	6.33% (*n* = 10)
	Dentistry	3.80% (*n* = 6)
	Public health	3.16% (*n* = 5)
	Midwifery	2.53% (*n* = 4)
	Other fields	6.96% (*n* = 11)
Prior volunteering experience	YES	74.05% (*n* = 117)
	NO	25.95% (*n* = 41)
Volunteering placement	Inpatient health facility (hospital/nursing home/quarantine facility)	42.40% (*n* = 67)
(more than one answer allowed)	Hospital checkpoint	31.65% (*n* = 50)
	Home, student hostel, Chamber of Physicians or other field work not directly at health facility	20.25% (*n* = 32)
	Outpatient clinic	13.92% (*n* = 22)
	Emergency service	13.29% (*n* = 21)
	Laboratory or drive thru	12.02% (*n* = 19)
	Sanitary and Epidemiological Inspection office	6.96% (*n* = 11)
	Pharmacy	1.90% (*n* = 3)
Patient contact	YES	65.19% (*n* = 103)
	NO	34.81% (*n* = 55)
Volunteering responsibilities	Taking patients' temperature	51.27% (*n* = 81)
(more than one answer allowed)	Taking patients' medical history face to face	46.84% (*n* = 74)
	Transport or logistics of patients, equipment or supplies	36.07% (*n* = 57)
	Patient triage	35.44% (*n* = 56)
	Patient care or support	29.75% (*n* = 47)
	Documentation (reports, analyses, contact tracing)	27.85% (*n* = 44)
	Taking patients' medical history on the phone	19.62% (*n* = 31)
	Informational and educational activities	16.45% (*n* = 26)
	Supporting healthcare workers with everyday tasks or helping to provide them with personal protective equipment	10.13% (*n* = 16)
	Helping at laboratories	8.86% (*n* = 14)
	Operating diagnostic equipment	3.80% (*n* = 6)

### Conditions and Safety

With reference to the conditions and safety of their service, most of the respondents reported that they had necessary equipment/tools (93.04%; *n* = 147) and PPE (personal protective equipment; 89.24%; *n* = 141), were able to take rest breaks while working (92.41%; *n* = 146), had access to technical support (88.61%; *n* = 140), and had received induction training (79.11%; *n* = 125). However, only 38.61% (*n* = 61) said they had access to psychological support when needed. We checked if there were any differences regarding SVP conditions between those volunteers who declared that they had direct contact with patients and those who did not. The respondents who had such contact more often reported having access to PPE, but less often – having access to psychological support and being able to take a rest (see Table 2).

**Table 2:** Reported conditions and safety of volunteering among the survey respondents with and without contact with patients (*n* = 158).

	**Had contact with patients 65.19% (*****n*** **=** **103)**			**Did not have contact with patients 34.81% (*****n*** **=** **55)**			***p***
I have received induction training to help me perform my tasks	YES	78.64%	(*n* = 81)	YES	80.00%	(*n* = 44)	*p* = 0.84131
	NO	21.36%	(*n* = 22)	NO	20.00%	(*n* = 11)	
I have the equipment and tools that are necessary for my work	YES	93.20%	(*n* = 96)	YES	92.73%	(*n =* 51)	*p* = 0.91072
	NO	6.80%	(*n* = 7)	NO	7.27%	(*n =* 4)	
I have the necessary personal protective equipment (face masks, gloves)	YES	93.20%	(*n =* 96)	YES	81.82%	(*n =* 45)	*p* = 0.02780
	NO	6.80%	(*n =* 7)	NO	18.18%	(*n =* 10)	
When necessary, I receive technical support (training, information materials, expert advice)	YES	86.41%	(*n =* 89)	YES	92.73%	(*n =* 51)	*p* = 0.23365
	NO	13.59%	(*n =* 14)	NO	7.27%	(*n =* 4)	
I have access to psychological support, when needed	YES	32.04%	(*n =* 33)	YES	50.91%	(*n =* 28)	*p* = 0.02029
	NO	67.96%	(*n =* 70)	NO	49.09%	(*n =* 27)	
I can take a rest break (to have a drink or meal)	YES	89.32%	(*n =* 92)	YES	98.18%	(*n =* 54)	*p* = 0.04519
	NO	10.68%	(*n =* 11)	NO	1.82%	(*n =* 1)	

Our student interviewees (SIs) expressed generally positive opinions on the safety of the project. One interviewee (SI.1) particularly appreciated expert support from the personnel of the beneficiary institution, who were “very friendly, very helpful.” Another (SI.4) said, “we were well-looked after.”

### Costs and Benefits

Time and the resulting learning-volunteering tensions were the only costs we asked about in the survey. The mean time of service among our participants was 39.5 days and the mean time spent volunteering was 22.04 h per week. 60.76% (*n* = 96) of the respondents stated that SV did not clash with the remote learning at the university. Two out of four student interviewees (SIs) said that lecturers respected the rule of postponing assessment for student volunteers (SI.1, SI.2), but the other two (SI.3, SI.4) noticed that violations, although rare, did happen. Three SIs (SI.3, SI.1, SI.2) said that participation was not too much of a burden in terms of time thanks to flexible volunteering schedules. One SI (SI.4) noticed that the beginnings were hard, with “too few people to fill the roster.” Three SIs (SI.1, SI.2, SI.4) remarked that the SVP was associated with a different kind of cost: stress. Its primary source was contacts with “misinformed” (SI.1, SI.2), “demanding” (SI.1) or even “aggressive” (SI.4) patients. Two SIs (SI.1, SI.4) felt stressed by the overburdened health system and the resulting shortages of human resources.

We were also interested if the volunteers noticed any project benefits, and educational benefits in particular. Generally speaking, 65.82% (*n* = 104) of the respondents agreed or strongly agreed with the statement *Volunteering allows me to learn new interesting things*. A majority noticed learning primarily soft skills: social (86.08%; *n* = 136), organisational (78.48%; *n* = 124) and stress management ones (68.99%; *n* = 109). Also 3 in 4 of our SIs (SI.1, SI.2, SI.4) said that volunteering honed their skills of communicating under stress. They emphasised that opportunities of learning communication during authentic patient – health professional contacts were scarce in regular university education. On the other hand, only 40.51% (*n* = 64) of the survey respondents noticed the development of their medical skills, even though 72.78% (*n* = 115) agreed or strongly agreed with the statement that volunteering was good for their future professional development. The interviews indicate a possible reason for that: 3 out of 4 of the SIs mentioned that the service provided them with an inside view of the health system and of the difficulties connected with pandemic response. They said how eye-opening it was to see “how the system really works, how much time and effort everything takes” (SI.3), “how the system responds to the pandemic” (SI.4), and that “there are so many shortages in the system” (SI.1). One person “worked like a regular employee” (SI.1). Another felt “as if we were sent to war” and admitted that “patients didn't go easy on us” (SI.4). According to SI.1, volunteering required more self-reliance than a regular internship.

Another area where the SVP proved beneficial was community links it created: making new contacts was among the most frequently observed benefits (82.28%; *n* = 130). One of our interviewees (SI.3) stressed that the project was one of the few opportunities for interprofessional collaboration offered by PUMS, and a unique chance for the students of different health professions to “leave their own castes” and appreciate skills of other specialties.

**Figure 1:**
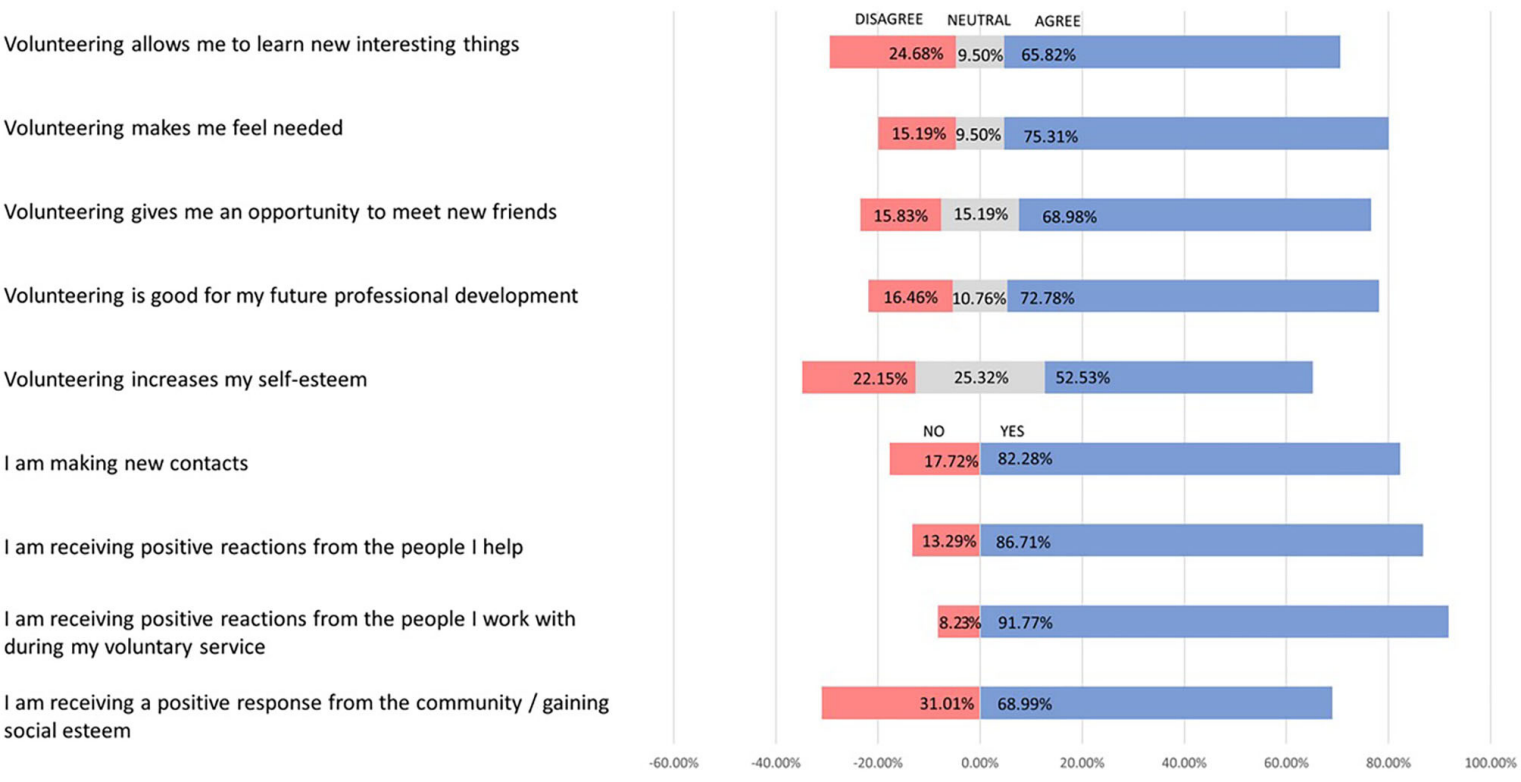
Reported benefits of volunteering among the survey respondents (*n* = 158).

One more aspect of the positive impact of the Poznań project on volunteers was the fact that it made them feel useful for the community: they felt needed (75.31%; *n* = 119) and believed that skills might be of use to the community (77.85%; *n* = 123). Moreover, they received positive reactions from colleagues (91.77%; *n* = 145) and beneficiaries (86.71%; *n* = 137). When we asked our interviewees, they said, “we were there when people needed us” (SI.3), “we were a real help, indispensable” (SI.4), “when we didn't know what to do with ourselves, we were able to help, in an organised way” (SI.2). Please see Figure 1 for details of the reported benefits of volunteering (Figure 1).

Next, we compared the importance of the tangible personal benefit (internship credit) and the intangible normative benefits among the survey respondents. 95.57% (*n* = 151) of them agreed or strongly agreed with the statement *I think it is important to help others*, and 77.85% (*n* = 123) – with *I believe that my skills may be of use to the community at this difficult time*. In contrast, only 26.58% (*n* = 42) of the respondents agreed or strongly agreed with the statement *I have decided to volunteer mostly to receive a credit for internship*. The latter group significantly less often reported learning new social skills (*p* = 0.00049), organisational skills (*p* = 0.00148), and skills of dealing with stressful/difficult situations (*p* = 0.00105) than those respondents who disagreed or strongly disagreed (see Table 3).

**Table 3:** Reported influence of volunteering on skills among the survey respondents grouped by attitude to internship credit as incentive (*n* = 158).

	**I have decided to volunteer mostly to receive a credit for internship**									
**I am learning new…**	**AGREE 26.58% (*****n=*** **42)**			**DISAGREE 62.66% (*****n=*** **99)**			**NEUTRAL 10.76% (*****n=*** **17)**			***p***
Medical skills	YES	68.18%	(*n =* 15)	YES	42.42%	(*n =* 42)	YES	41.18%	(*n =* 7)	0.45779
	NO	31.82%	(*n =* 27)	NO	57.58%	(*n =* 57)	NO	58.82%	(*n =* 10)	
Social skills	YES	69.05%	(*n =* 29)	YES	91.92%	(*n =* 91)	YES	94.12%	(*n =* 16)	0.00049
	NO	30.95%	(*n =* 13)	NO	8.08%	(*n =* 8)	NO	5.88%	(*n =* 1)	
Organisational skills	YES	61.90%	(*n =* 26)	YES	85.86%	(*n =* 85)	YES	76.47%	(*n =* 13)	0.00148
	NO	38.10%	(*n =* 16)	NO	14.14%	(*n =* 14)	NO	23.53%	(*n =* 4)	
Skills of dealing with stressful/difficult situations	YES	50.00%	(*n =* 21)	YES	77.78%	(*n =* 77)	YES	64.71%	(*n =* 11)	0.00105
	NO	50.00%	(*n =* 21)	NO	22.22%	(*n =* 22)	NO	35.29%	(*n =* 6)	

While only a fourth of the survey respondents seem driven by the academic credit, two of our interviewees reported a noticeably bigger influx of new SVs after the decision to grant the credit was announced. According to SI.3, “the idea of SVP was spoiled by adding the internship credit.” Another interviewee (SI.4) stated that the internship-motivated students “can't be called real volunteers” and that the decision “distorted the idea of volunteering.” Both said that the students who were motivated by gaining a credit “botched up their service hours.” At the same time, SI.4 and SI.2 pointed out that the decision was necessary because it partly ameliorated the suspension of practical classes and did not place additional burden on volunteers by requiring them to take internships on top of their service. Three SIs (SI.3, SI.2, and SI.4) said that replacing internships with volunteering was an emergency solution, and that volunteering in general was unfeasible as a required curricular activity but perhaps workable as an elective or selective course. SI.1's volunteering experience was “actually better than internships,” but “not all volunteers were so lucky.”

Finally, there was one benefit we analysed in more detail: the impact of the SVP on students' feelings about their study choices. It turned out that for 60.76% (*n* = 96) of the survey respondents, it was volunteering that helped them to make sure that they had made the right choice of the field of study. This group also significantly more often noticed some intangible benefits of volunteering (see Table 4).

**Table 4:** Reported benefits of volunteering among the survey respondents who found that volunteering confirmed their study choices compared with those who did not find that (*n* = 158).

	**I have found that I made the right choice of the field of study**						
	**YES 60.76% (*****n=*** **96)**			**NO 39.24% (*****n=*** **62)**			***p***
Volunteering allows me to learn new interesting things	YES	80.21%	(*n =* 77)	YES	43.55%	(*n =* 27)	<0.00001
	NO	12.50%	(*n =* 12)	NO	43.55%	(*n =* 27)	
	UNDECIDED	7.29%	(*n =* 7)	UNDECIDED	12.90%	(*n =* 8)	
Volunteering makes me feel needed	YES	89.58%	(*n =* 86)	YES	53.23%	(*n =* 33)	0.00001
	NO	6.25%	(*n =* 6)	NO	29.03%	(*n =* 18)	
	UNDECIDED	4.17%	(*n =* 4)	UNDECIDED	17.74%	(*n =* 11)	
I believe that my skills may be of use to the community at this difficult time	YES	87.50%	(*n =* 84)	YES	62.90%	(*n =* 39)	0.00016
	NO	4.17%	(*n =* 4)	NO	22.58%	(*n =* 14)	
	UNDECIDED	8.33%	(*n =* 8)	UNDECIDED	14.52%	(*n =* 9)	
Volunteering gives me an opportunity to meet new friends	YES	70.83%	(*n =* 68)	YES	66.13%	(*n =* 41)	0.18598
	NO	12.50%	(*n =* 12)	NO	20.97%	(*n =* 13)	
	UNDECIDED	16.67%	(*n =* 16)	UNDECIDED	12.90%	(*n =* 8)	
I think it is important to help other people	YES	95.83%	(*n =* 92	YES	95.16%	(*n =* 59)	0.75319
	NO	1.04%	(*n =* 1)	NO	1.61%	(*n =* 1)	
	UNDECIDED	3.13%	(*n =* 3)	UNDECIDED	3.23%	(*n =* 2)	
Volunteering is good for my future professional development	YES	87.50%	(*n =* 84)	YES	50.00%	(*n =* 31)	<0.00001
	NO	5.21%	(*n =* 5)	NO	33.87%	(*n =* 21)	
	UNDECIDED	7.29%	(*n =* 7)	UNDECIDED	16.13%	(*n =* 10)	
Volunteering increases my self-esteem	YES	64.58%	(*n =* 62)	YES	33.87%	(*n =* 21)	0.00001
	NO	11.46%	(*n =* 11)	NO	38.71%	(*n =* 24)	
	UNDECIDED	23.96%	(*n =* 23)	UNDECIDED	27.42%	(*n =* 17)	
I am learning new medical skills	YES	51.04%	(*n =* 49)	YES	24.19%	(*n =* 15)	0.00079
	NO	48.96%	(*n =* 47)	NO	75.81%	(*n =* 47)	
I am learning new social skills	YES	90.63%	(*n =* 87)	YES	79.03%	(*n =* 49)	0.03985
	NO	9.38%	(*n =* 9)	NO	20.97%	(*n =* 13)	
I am learning new organisational skills	YES	87.50%	(*n =* 84)	YES	64.52%	(*n =* 40)	0.0006
	NO	12.50%	(*n =* 12)	NO	35.48%	(*n =* 22)	
I am learning new skills of dealing with stressful or difficult situations	YES	79.17%	(*n =* 76)	YES	53.23%	(*n =* 33)	0.00058
	NO	20.83%	(*n =* 20)	NO	46.77%	(*n =* 29)	
I am making new contacts	YES	87.50%	(*n =* 84)	YES	74.19%	(*n =* 46)	0.03245
	NO	12.50%	(*n =* 12)	NO	25.81%	(*n =* 16)	
I am receiving positive reactions from the people I help	YES	91.67%	(*n =* 88)	YES	79.03%	(*n =* 49)	0.02236
	NO	8.33%	(*n =* 8)	NO	20.97%	(*n =* 13)	
I am receiving positive reactions from the people I work with during my voluntary service	YES	94.79%	(*n =* 91)	YES	87.10%	(*n =* 54)	0.08566
	NO	5.21%	(*n =* 5)	NO	12.90%	(*n =* 8)	
I am receiving a positive response from the community/gaining social esteem	YES	76.04%	(*n =* 73)	YES	58.06%	(*n =* 36)	0.01706
	NO	23.96%	(*n =* 23)	NO	41.94%	(*n* = 26)	

## Discussion

### Main Findings and Comparison With Other Studies

The first area we analysed was the volunteers' perceptions on the conditions and safety of the project. Among similar projects carried out globally in response to the COVID-19 pandemic, we found one whose participants were asked to assess safety of their service. 74.6% of the 65 medical students who were involved in testing healthcare employees through the Johannes Gutenberg-University in Mainz, Germany, felt well-protected during service, although only 12.19% felt well-prepared for their tasks (16). In our study, students' perceptions on safety sometimes differed from what was guaranteed to them under their contracts with beneficiary institutions. Alarmingly, nearly 7% of the respondents who had contact with patients reported that they did not have the necessary PPE. It is also worrying that over 20% of the respondents said they had not received induction training, and around 11% did not have technical support (including training, information materials, and expert advice). On the one hand, these numbers reveal the weakness of the health system during the crisis. On the other hand, they may result from the fact that the project was organised *ad-hoc*, without sufficient material and human resources to guarantee proper safety and arrange a training and mentoring framework. A solution to that could be using fast-track remote preparatory courses: either tailored ones developed by universities for their own students, as it happened at universities in the USA (20), Denmark (17), and Switzerland (13), or courses already prepared at different universities and made available to other medical schools, as was the case with Harvard Medical School, USA (19). Our findings also suggest that it is crucial to ask student volunteers about the safety and conditions of volunteering in order to discover and resolve any discrepancies between policy and practice.

In the area of costs related to SVP participation, we found that nearly 40% of the survey respondents felt that the service clashed with their learning responsibilities, and 2 out of 4 SIs reported (though infrequent) difficulties arranging postponed assessment with individual lecturers. This indicates that the university authorities failed to properly explain or communicate the new assessment rule to the faculty, or just failed to enforce it rigorously enough. Another cost, which we did not ask about in the survey but which was mentioned during the interviews, was stress. Coupled with the absence of psychological support reported by over 60% of the respondents, this indicates that insufficient care was taken to ensure volunteers' well-being. Well-being assistance does not always require substantial institutional outlays: it can be provided by volunteers themselves, as it happened at the Columbia University in the USA (11, 18).

When analysing benefits connected with the service, we were particularly interested in the educational benefits. The majority of our participants reported learning new skills, and so did participants of other COVID-19 SVPs. The participants of the Mainz study noticed the positive impact of volunteering: 68.3% said they had learned new skills, and 81.0% said they had improved their existing skills due to the commitment (16). More specific questions about skills were asked to medical student volunteers from the Reno School of Medicine, USA, who staffed a COVID-19 hotline for rural communities. The students reported 75 to 366.67% improvement in comfort with various tasks connected with audio-only patient contact (20). Another detailed account of educational benefits of volunteering was collected in an open-text survey among students from the University of Nebraska College of Public Health, USA, supporting COVID-19 public education and efforts of a local epidemiology team. The benefits listed by the students included such valuable skills as real-time data collection, social media monitoring, and effective communication with communities and public health organisations (22). Since our student volunteers performed a wide range of tasks, we asked them only about broad categories of skills. The survey results suggest that volunteering positively influenced students' soft skills (especially social ones). In addition, our respondents widely recognised social benefits of volunteering: feeling useful to and being appreciated by the community. The importance of this aspect was also highlighted at the Paulista School of Medicine in São Paulo, Brasil. Its SVP, inspired by medical students, generated enthusiasm, a sense of purpose and gratitude from beneficiaries. As the authors put it, “the project's main outcome is the moment of solidarity it has started, involving more and more participants and impacting far beyond the hospital walls” (10).

The interviews with our students indicated that volunteering also enhanced their understanding of how the health system works as a multidisciplinary whole. In fact, the Poznań project seems to be among the few interprofessional COVID-19 SVPs described in the latest literature, partly due to the fact that some medical schools offer only medical programmes. A large-scale multidisciplinary SVP was initiated by the Columbia University Irving Medical Center in New York, USA; it soon turned into a national initiative (11, 18). The project website stresses that collaboration should be interprofessional “to promote students learning with, from, and about each other through their service” (34). Another multidisciplinary project was launched at the University of Nebraska Medical Center in Omaha, USA, where a number of student-led volunteering initiatives were integrated with the activities of an emergency preparedness and response institution. The result was a “campus-wide, interprofessional effort with a diverse volunteer pool,” which replaced “siloed volunteer structures designed by and for specific health professions” (14).

It should be noted that the area where our students noticed the least improvement thanks to the SVP was learning new medical skills (40.51%). It is hardly surprising. The decision to treat the SVP as an equivalent of regular internships was understandable, but problematic. Understandable, because it meant that student volunteers thus gained a chance to hone at least some practical skills during the lockdown. And problematic, because volunteering did not always teach them the skills required by the curriculum and did not offer many opportunities for mentoring, feedback and reflection. It was caused by the *ad-hoc* character of the initiative and the resulting absence of a pre-existing conceptual model and organisational framework.

For example, the concept of our SVP was largely determined by the fact that student volunteers were initially treated as emergency workforce rather than stakeholders in their own right and with their own needs. This might have been caused by the fact that representatives of particular beneficiary institutions did not participate in the works of the SVP coordinating team. Closer collaboration of all the stakeholders might have enabled the university authorities and the students themselves to promote a more balanced project concept, with a bigger focus on students' learning needs.

The available literature suggests that the pandemic SVPs which offered the most to students in terms of educational benefits were those organised at universities using well-tried models of experiential learning and having long-standing traditions of doing so. Such models are often based on community-campus partnerships and incorporate appropriate mentoring and reflection mechanisms to support student learning. One example was the SVP at Columbia, USA, where a service-learning model was used to ensure close collaboration between students and faculty members and to bridge community needs with student learning objectives (11, 18). The university required its student volunteers to take part in reflection and debriefing sessions (18). Aalborg University, Denmark, also decided to match the pandemic emergency with the learning objectives of undergraduate medical curriculum. As the university had relied on problem-based learning since 1974 (35), it managed to make its COVID-19 SVP operational within 2 weeks thanks to close collaboration between the hospital, the university, and students' organisations. A new portfolio was constructed for student volunteers for assessment purposes (17). At British Columbia, Canada, volunteering was partly incorporated into FLEX, the university's flexible programme of individual scholarly projects (12). The projects are guided by supervisors, who help students develop project activities, provide direction and mentorship during students' engagement, and assess their performance (36). It can be concluded that volunteer service is easier to incorporate also into curricula which include individual learning opportunities.

All the three models described above assumed granting academic credits to student volunteers and treating SVPs as curricular activities. Such a practice is well-grounded and seems to work well at universities around the world (3, 37). It should be noted, however, that formalising and mandating voluntary activities is a sensitive issue. A formal incentive or requirement to volunteer may encourage participation (38, 39), but might sometimes be perceived as an imposition taking away students' time and may consequently reduce their internal motivation to volunteer, satisfaction levels, university commitment, and future intentions to volunteer (40–42). Some of these consequences might have arisen in the Poznań SVP. Although the internship credit proved to be a powerful magnet to our volunteers, some student interviews suggest that the volunteers it attracted were not as conscientious as the others. Those survey respondents who were incentivised by the internship credit also significantly less often reported learning new soft skills while volunteering. The reasons would require more in-depth research, but one possible explanation could be decreased motivation and satisfaction due to the presence of an academic credit perceived as a form of external constraint. If this is the case, the experiences from educational systems with mandatory volunteering show that it is possible to mitigate the negative effect of requirements by increasing volunteers' perceptions of intrinsic motivation (e.g., by demonstrating how volunteering fits with students' own goals) and autonomy (e.g., by allowing volunteers to choose placements matching their primary motivations) (43, 44).

Finally, turning volunteering into a curricular activity in post-communist countries of Eastern and Central Europe may be hindered by specific cultural and social factors. According to Preradović and Mažeikienė, these societies have deeply embedded mistrust of volunteering through public organisations, which were highly regulated in the communist times. At the same time, they have well-developed informal social networks which tend to be favoured over formal structures (45). This is corroborated by the latest survey by Statistics Poland, which found that 30.9% of the respondents had been involved in informal volunteering within 4 weeks prior to the survey, while only 8.5% had engaged in formal volunteering (46). This means that building trust in formal volunteering within these societies may take time and may require paying special attention to organisational and procedural transparency. As Preradović and Mažeikienė pointed out, the faculty in these countries are often underpaid and overworked compared to their peers at universities in Western Europe and the USA. Consequently, they may not have enough time and motivation to seek innovations in curricular design (45). Therefore, it would be wise to use the experiences of predecessors and build partnerships with universities with longer traditions of experiential learning. It must be stressed, however, that such solutions are culture-sensitive and should not be simply transplanted from the countries where they originated and whose civic cultures they match. While foreign models should be explored to avoid reinventing the wheel and duplicating efforts, local challenges and determinants must be identified. Thus, to guide future practice, in-depth research is required to determine the expectations and preferences with regard to SV among different stakeholders: students, faculty members, and beneficiary institutions.

### Strengths and Limitations

The strength of our study is the fact that it presents SV experiences in a country without traditions of infusing volunteering into university curricula. On the one hand, volunteering of health professions students in Poland meant more than just filling the gaps in the understaffed health system, which seemed to be the case in some African countries (15). On the other hand, it fell short of being an effective educational tool, which was the case in the countries where formalised SV has longer traditions. As such, the Polish SVP can be quite instructive to medical schools in communities with similar characteristics.

A limitation of this study was the use of a non-standardised questionnaire as our primary research tool. We asked the questions that, in our opinion, best suited the circumstances. Next, the study did not investigate the impact of the SVP on the community and the perspectives of all the stakeholders: patients, faculty members, and staff of beneficiary institutions. Finally, we did not fully explore volunteers' satisfaction with the project, their views on its weaknesses, and opinions on the viability of volunteering projects as future curricular activities.

## Conclusions

The 2020 COVID-19 pandemic taught us a few important lessons. Although it caused a serious disruption of health professions education, it also gave rise to a number of valuable SV initiatives around the world. It suggests that SV is a viable solution for such disruptions and that the patient contact opportunities it offers may well complement remote or simulated learning. However, if SV is to effectively support health professions education, it requires a systemic approach which includes:

(1) promoting the idea of SV by presenting its benefits in order to tap and fuel volunteers' enthusiasm;
(2) using a well-thought-out and transparent organisational framework to balance the needs and channel the efforts of multiple players;
(3) maximising the educational benefits of SV by introducing opportunities for mentoring and reflection;
(4) maximising volunteers' safety by ensuring induction training, sufficient equipment and psychological support;
(5) exploring expectations of all stakeholders in order to develop and implement culture-sensitive solutions.

Our SV experiences showed that health professions students are willing to volunteer in large numbers and appreciate the practicality of volunteering, which often helps to confirm their career choices. We believe that such initiatives can become an impulse for implementing SV as a complementary element of health professions education in Poland and other countries of Central and Eastern Europe, where formal volunteering is still approached with reserve. Our findings as well as the available literature lead us to conclude that such countries could use the best practices from more experienced educational systems to build a strong SV organisational framework, but at the same time should make sure that formalisation of volunteering does not reduce the perceived value of volunteering in the eyes of the participants.

To achieve the first goal, a university should consider setting up a permanent student volunteering committee that would develop appropriate procedures for launching and running SVPs during and between emergencies such as epidemics. In non-emergency periods, a small number of SVPs could be made available to students on a regular basis. In emergency times, the procedures would allow for a swift and smooth launch of additional SVPs to support the understaffed healthcare system. The committee, acting as a liaison between different stakeholders, would determine, record and match the personnel needs of healthcare facilities with students' educational needs defined in curricula.

To achieve the second goal, a university should try to maximise and capitalise on the voluntary nature of SV so as to increase the intrinsic motivation for participation. One way to accomplish that is to build and emphasise the educational potential of SV, for instance through feedback and debriefing sessions with peers and mentors, or through official and informal communications presenting educational benefits of volunteering. Another way is to devise a model of volunteering based on student autonomy, with as many points of choice as possible. For example, an undergraduate health professions student could choose from the following forms of participation in volunteering:

(1) **internship SVPs as an alternative to regular internships**. In times of emergency, students could choose to participate in internship SVPs or wait until regular internships are resumed. Since each course and year has specific learning outcomes to be achieved during internships, this form of participation would probably be the most suitable for first- or second-year students required to develop non-specialist competencies or soft skills. Placements might involve registering patients, managing patient flow, doing administrative jobs or epidemiological tasks. A student would be granted a credit after completing the required number of hours and reflecting on them in an internship report, and given a mark based on performance assessments made jointly by a supervisor from a host facility and an internship coordinator from the university;
(2) **elective SVPs**. Each student is required to choose one or more elective courses to gain a specific number of credits in a given year, but electives are not as strictly regulated as internships in terms of learning outcomes. As such, elective SVPs would be available to a wide group of students provided that they meet entry requirements set by healthcare facilities. Taking an elective SVP would give a student an opportunity to choose from a number of different placements, some of them offering insights into unfamiliar settings or novel tasks. A credit would be granted after completing a pre-defined number of hours and submitting an assignment required by an elective coordinator;
(3) **non-credit SVPs**. This form of participation could be offered to all students willing to volunteer just for the sake of volunteering. It could also be open to the students who completed the required number of hours as part of their credited volunteering but wish to keep helping others. The participants would be free to choose the length of their service as well as their placement on condition that they met entry requirements.

The implementation of such a model may present a big organisational challenge. However, with the first steps already taken by numerous universities during the COVID-19 pandemic, continuing along this road could soon pay off and benefit students and patients alike.

## Data Availability Statement

The raw data supporting the conclusions of this article will be made available by the authors, without undue reservation.

## Ethics Statement

Ethical review and approval was not required for the study on human participants in accordance with the local legislation and institutional requirements. Written informed consent for participation was not required for this study in accordance with the national legislation and the institutional requirements.

## Author Contributions

EC, RS, AL, and JW: conceptualization. EC, MK, and MC: methodology. DB, RS, AL, and EC: acquisition of data. EC, MC, MN, BG, and AL: analysis of data. EC, RS, AL, BG, MK, DB, MN, MC, and JW: writing, review, and editing. RS and AL: project administration. EC and JW: supervision. All authors gave final approval of the paper and agreed to be accountable for all aspects of the work in ensuring that questions related to the accuracy or integrity of any part of the work are appropriately investigated and resolved.

## Conflict of Interest

The authors declare that the research was conducted in the absence of any commercial or financial relationships that could be construed as a potential conflict of interest.

## Abbreviations

PPE: personal protective equipment
PUMS: Poznan University of Medical Sciences
SIs: student interviewees
SV: student volunteering
SVP: student volunteering project.

## References

[1] Paull M Scott R MacCallum J Walker G Omari M Young S University student volunteering: what's in a name? Third Sect Rev. (2015) 21:49–74. Available online at: https://search.informit.com.au/documentSummary;dn=710442058548381;res=IELHSS (accessed January 10, 2021).

[2] Furco A Service-learning: a balanced approach to experiential education. In: Taylor B, editor. Expanding Boundaries: Serving and Learning. Washington, DC: Corporation for National Service (1996). p. 2–6.

[3] Aramburuzabala P McIlrath L Opazo H eds. Embedding Service Learning in European Higher Education: Developing a Culture of Civic Engagement. London: Routledge (2019).

[4] Ustawa z dnia 24 kwietnia 2003 r. o działalności pożytku publicznego i o wolontariacie [The public benefit and volunteer work act of 24 April 2003]. J Laws 2003. (2003) 873.

[5] Ministerstwo Zdrowia. Wolontariat. Available online at: http://www.archiwum.mz.gov.pl/system-ochrony-zdrowia/kadra-medyczna-i-ksztalcenie/wolontariat/ (accessed October 12, 2020).

[6] World Health Organization WHO Director-General's Opening Remarks at the Media Briefing on COVID-19 - 11 March 2020. (2020). Available online at: https://www.who.int/dg/speeches/detail/who-director-general-s-opening-remarks-at-the-media-briefing-on-covid-19-−11-march-2020 (accessed September 4, 2020).

[7] Gordon M Patricio M Horne L Muston A Alston SR Pammi M. Developments in medical education in response to the COVID-19 pandemic: a rapid BEME systematic review: BEME Guide No. 63. Med Teach. (2020) 42:1202–15. 10.1080/0142159X.2020.1807484 32847456

[8] Dedeilia A Sotiropoulos MG Hanrahan JG Janga D Dedeilias P Sideris M. Medical and surgical education challenges and innovations in the COVID-19 era: a systematic review. In Vivo. (2020) 34:1603–11. 10.21873/invivo.11950 32503818 PMC8378024

[9] Ayoub P Chang DD Hussein N Stewart K Wise A Malik I. Medical student mobilization during a pandemic: the ochsner clinical school response to COVID-19. Ochsner J. (2020) 20:146–50. 10.31486/toj.20.0069 32612468 PMC7310173

[10] Mendes Chiloff D Muniz de Freitas V Borges Cardin N Bianchi Matiuzzi C Juliano Perfeito JA Inoue Sato E Volunteering in medical school during the pandemic: a solution for teaching. MedEdPublish. (2020) 9:114 10.15694/mep.2020.000114.1 PMC10702680 38073813

[11] Edelman DS Desai UA Soo-Hoo S Catallozzi M. Responding to hospital system and student curricular needs: COVID-19 student service corps. Med Educ. (2020) 54:853–4. 10.1111/medu.14243 32418240 PMC7276917

[12] Haines MJ Yu ACM Ching G Kestler M. Integrating a COVID-19 volunteer response into a Year-3 MD curriculum. Med Educ. (2020) 54:960–1. 10.1111/medu.14254 32438453 PMC7280601

[13] Klasen JM Meienberg A Nickel C Bingisser R. SWAB team instead of SWAT team: medical students as a frontline force during the COVID-19 pandemic. Med Educ. (2020) 54:860. 10.1111/medu.14224 32403176 PMC7272889

[14] Kratochvil TJ Khazanchi R Sass RM Caverzagie KJ. Aligning student-led initiatives and Incident command system resources in a pandemic. Med Educ. (2020) 54:1183–4. 10.1111/medu.14265 32502292 PMC7300600

[15] Mkenda V Woolhouse M Mutapi F Banda G Recruiting students for the COVID-19 emergency response: lessons from eight African countries. AAS Open Res. (2020) 3:42 10.12688/aasopenres.13115.1

[16] Müller L Heymanns M Harder L Winter J Gehring S Düber C Medical students' commitment during the SARS-CoV-2 pandemic: preparedness, motivation, and impact on students' skills. Res Square. (2020). 10.21203/rs.3.rs-37069/v1 [preprint].

[17] Rasmussen S Sperling P Poulsen MS Emmersen J Andersen S. Medical students for health-care staff shortages during the COVID-19 pandemic. Lancet. (2020) 395:e79–80. 10.1016/S0140-6736(20)30923-5 32334649 PMC7180031

[18] Rupley D Grilo SA Kondragunta S Amiel J Matseoane-Peterssen D Catallozzi M. Mobilization of health professions students during the COVID-19 pandemic. Semin Perinatol. (2020) 44:151276. 10.1016/j.semperi.2020.151276 32798093 PMC7373033

[19] Soled D Goel S Barry D Erfani P Joseph N Kochis M. Medical student mobilization during a crisis: lessons from a COVID-19 medical student response team. Acad Med. (2020) 95:1384–7. 10.1097/ACM.0000000000003401 32282373 PMC7188031

[20] Carson S Peraza LR Pucci M Huynh J. Student hotline improves remote clinical skills and access to rural care. PRiMER. (2020) 4:22. 10.22454/PRiMER.2020.581719 33111049 PMC7581206

[21] Office EE Rodenstein MS Merchant TS Pendergrast TR Lindquist LA. Reducing social isolation of seniors during COVID-19 through medical student telephone contact. J Am Med Dir Assoc. (2020) 21:948–50. 10.1016/j.jamda.2020.06.003 32674825 PMC7274632

[22] Chengane S Cheney A Garth S Medcalf S. The COVID-19 response in Nebraska: How students answered the call. Prev Chronic Dis. (2020) 17:200269. 10.5888/pcd17.200269 32790607 PMC7458111

[23] Poland Coronavirus: Worldometer Worldometers.info Available online at: https://www.worldometers.info/coronavirus/country/poland/ (accessed October 2, 2020).

[24] OECD. Health at a Glance 2019: OECD Indicators. Paris: OECD Publishing (2019).

[25] Solecka M Jeden medyk – jedno miejsce pracy. Med Prakt. (2020). Available online at: https://www.mp.pl/kurier/231361,jeden-medyk-jedno-miejsce-pracy (accessed September 7, 2020).

[26] Rozporządzenie Ministra Zdrowia z dnia 28 kwietnia 2020 r. w sprawie standardów w zakresie ograniczeń przy udzielaniu świadczeń opieki zdrowotnej pacjentom innym niz z podejrzeniem lub zakazeniem wirusem SARS-CoV-2 przez osoby wykonujace zawód medyczny majace bezpośredni kontakt z pacjentami z podejrzeniem lub zakazeniem tym wirusem [Regulation issued by the Minister of Health on 28 April 2020 regarding the standards of limitations related to the provision of healthcare to patients not infected or suspected of infection with the SARS-CoV-2 virus by health professionals having direct contact with patients infected or suspected of infection with the virus]. J Laws 2020. (2020) 775.

[27] Ministerstwo Nauki i Szkolnictwa Wyzszego List do Rektorów Uczelni Medycznych w Sprawie Wolontariatu Zwiazanego z Zagrozeniem COVID-19. (2020). Available online at: https://www.gov.pl/web/nauka/list-do-rektorow-uczelni-medycznych-w-sprawie-wolontariatu-zwiazanego-z-zagrozeniem-covid-19 (accessed September 14, 2020).

[28] Rozporządzenie Ministra Nauki i Szkolnictwa Wyzszego z dnia 11 marca 2020 r. w sprawie czasowego ograniczenia funkcjonowania niektórych podmiotów systemu szkolnictwa wyzszego i nauki w zwiazku z zapobieganiem, przeciwdziałaniem i zwalczaniem COVID-19 [Regulation issued by the Minister of Science and Higher Education on 11 March 2020 regarding temporary limitations on the functioning of selected higher education and scientific institutions imposed to prevent and counter COVID-19]. J Laws 2020 (2020) 405.

[29] Hustinx L Cnaan RA Handy F Navigating theories of volunteering: a hybrid map for a complex phenomenon. J Theory Soc Behav. (2010) 40:410–34. 10.1111/j.1468-5914.2010.00439.x

[30] Wilson J Volunteering. Annu Rev Sociol. (2000) 26:215–40. 10.1146/annurev.soc.26.1.215

[31] Chinman MJ Wandersman A The benefits and costs of volunteering in community organizations: review and practical implications. Nonprofit Volunt Sect Q. (1999) 28:46–64. 10.1177/0899764099281004

[32] Cnaan RA Handy F Wadsworth M Defining who is a volunteer: conceptual and empirical considerations. Nonprofit Volunt Sect Q. (1996) 25:364–83. 10.1177/0899764096253006

[33] Haski-Leventhal D Altruism and volunteerism: the perceptions of altruism in four disciplines and their impact on the study of volunteerism. J Theory Soc Behav. (2009) 39:271–99. 10.1111/j.1468-5914.2009.00405.x

[34] Vagelos College of Physicians and Surgeons. COVID-19 Student Service Corps (CSSC). Available online at: https://www.ps.columbia.edu/education/covid-19-student-service-corps-cssc/toolkit-new-chapters (accessed September 11, 2020).

[35] Aalborg University The Aalborg Model for Problem Based Learning. Available online at: https://www.en.aau.dk/about-aau/aalborg-model-problem-based-learning/ (accessed September 14, 2020).

[36] UBC Faculty of Medicine/MedNet FLEX. Info for Activity Supervisors. Available online at: https://mednet.med.ubc.ca/Teaching/FLEX/Pages/Info-for-Activity-Supervisors.aspx (accessed October 16, 2020).

[37] Stewart T Wubbena Z. An overview of infusing service-learning in medical education. Int J Med Educ. (2014) 5:147–56. 10.5116/ijme.53ae.c907 25341224 PMC4212253

[38] Metz E Youniss J A demonstration that school-based required service does not deter - but heightens - volunteerism. Polit Sci Polit. (2003) 36:281–6. 10.1017/S1049096503002221

[39] Henderson A Brown SD Pancer SM Ellis-Hale K Mandated community service in high school and subsequent civic engagement: the case of the double cohort in Ontario, Canada. J Youth Adolesc. (2007) 36:849–60. 10.1007/s10964-007-9207-1

[40] Beehr TA Legro K Porter K Bowling NA Swader WM Required volunteers: Community volunteerism among students in college classes. Teach Psychol. (2010) 37:276–80. 10.1080/00986283.2010.510965

[41] Stukas AA Snyder M Clary EG The effects of “mandatory volunteerism” on intentions to volunteer. Psychol Sci. (1999) 10:59–64. 10.1111/1467-9280.00107

[42] Clary EG Snyder M The motivations to volunteer: theoretical and practical considerations. Curr Dir Psychol Sci. (1999) 8:156–9. 10.1111/1467-8721.00037

[43] Stukas AA Snyder M Clary EG. Understanding and encouraging volunteerism and community involvement. J Soc Psychol. (2016) 156:243–55. 10.1080/00224545.2016.1153328 27064177

[44] Henney SM Hackett JD Porreca MR Involuntary volunteerism: what happens when you require people to do good? J Serv High Educ. (2017)6:49–61. Available online at: https://journals.sfu.ca/jslhe/index.php/jslhe/article/view/126 (accessed January 10, 2021).

[45] Preradović NM MaŽeikiene N Service learning in post-communist countries. In: Aramburuzabala P McIlrath L Opazo H editors. Embedding Service Learning in European Higher Education: Developing a Culture of Civic Engagement. London: Routledge (2020). p. 180–195.

[46] Czerwiński M Kazanowska D Kazimierowska–Wasiołek M Knapp A Pragacz M eds. Wolontariat w 2016 r. Warszawa: Główny Urzad Statystyczny (2017).

