# Supplementary material for: Student Volunteering as a Solution for Undergraduate Health Professions Education: Lessons From the COVID-19 Pandemic
Source: Front Public Health. 2021 Jan 26;8:633888. doi: 10.3389/fpubh.2020.633888 (PMC7871007; doi:10.3389/fpubh.2020.633888)
Supplement: Supplementary file 1 [file Table_1.DOCX]

Supplementary File 1. The COVID-19 student volunteering survey

**COVID-19 VOLUNTEERING**

Dear Students,

We are conducting a survey among the volunteers who have decided to help during the COVID-19 pandemic caused by SARS-CoV-2. The survey is completely anonymous. It should take up to 10 minutes to answer all the questions.

Thank you for taking the time to complete this important questionnaire.

At the same time, we would like to express our appreciation for your attitude and active participation in the struggle with the effects of the COVID-19 pandemic.

Department of Preventive Medicine

Poznan University of Medical Sciences

ul. Święcickiego 6

60-781 Poznań

If you have any questions, please contact:

Ewelina Wierzejska, PhD

email: [ewierz@ump.edu.pl](mailto:ewierz@ump.edu.pl)

1. Your gender

Please select one answer.

- Female
- Male

1. Your age

Please complete ……………

1. Your field of study

Please select one answer.

- Medicine
- Dentistry
- Nursing
- Pharmacy
- Medical Laboratory Science
- Midwifery
- Paramedic Science
- Physiotherapy
- Dietetics
- Cosmetology
- Public Health
- Occupational Therapy
- Dental Technology
- Hearing Aid Audiology
- Biotechnology
- Pharmaceutical Engineering
- Forensic Science
- Radiography
- Optometry

1. Have you been a volunteer before?

Please select one answer.

- YES
- NO

1. If you have, please indicate the area of volunteer service (you may choose more than one answer):

Please tick all that apply.

- short-term volunteering at events (e.g. fundraising for such charities as the Great Orchestra of Christmas Charity or the Noble Gift)
- long-term volunteering in health care
- long-term welfare or social volunteering
- school volunteering projects
- international volunteering (e.g. at a work camp)
- long-term volunteering in humanitarian aid
- long-term volunteering in other areas

1. If you have been a volunteer before, please indicate how long.

Please select one answer.

- less than 1 year
- 1-2 years
- 3-4 years
- 5 years or more

1. How long have you been volunteering in response to the COVID-19 pandemic? Number of days:

Please complete ……………

1. How many hours a week do you spend volunteering in response to the COVID-19 pandemic? (if the number varies week by week, please provide a mean weekly number observed in a month)

Please complete ……………

1. Does your COVID-19 voluntary service clash with the current remote learning at the university?

Please select one answer.

- YES
- NO

1. Please indicate the place of your COVID-19 voluntary service (you may choose more than one answer):

Please tick all that apply.

- checkpoint at a hospital entrance
- hospital department / ward (please indicate which ward) ……………
- outpatient clinic
- university laboratory
- emergency service
- office of the Sanitary and Epidemiological Inspection
- pharmacy
- home or student hostel – remote (virtual / phone) volunteering
- other, please specify ……………

1. Please indicate your responsibilities related to the COVID-19 volunteering (you may select more than one answer):

Please tick all that apply.

- taking patients’ medical history face to face at a hospital, checkpoint, etc.
- taking patients’ medical history on the phone
- assistance at patient triage
- taking patients’ temperature
- logistic support or transport of patients, equipment or supplies
- support in the delivery of patient care or patient support
- helping at a laboratory
- operating diagnostic equipment
- clerical work (preparing reports and analyses, tracing contacts, etc.)
- helping healthcare workers with everyday tasks (babysitting, shopping, etc.)
- organising information and educational activities
- other, please specify ………………

1. What kind of contact with patients do you have?

Please tick all that apply.

- I have direct contact with SARS-CoV-2-infected patients and their families
- I have direct contact with quarantined patients and their families
- I have direct contact with patients with unknown SARS-CoV-2 status (undiagnosed and non-quarantined patients) and their families
- I do not have direct contact with patients

Please specify the working conditions during your COVID-19 voluntary service:

1. I have received induction training to help me perform my tasks.

Please select one answer.

- YES
- NO

1. I have the equipment and tools that are necessary for my work.

Please select one answer.

- YES
- NO

1. I have the necessary personal protective equipment (face masks, gloves).

Please select one answer.

- YES
- NO

1. When necessary, I receive technical support (training, information materials, expert advice).

Please select one answer.

- YES
- NO

1. I have access to psychological support, when needed.

Please select one answer.

- YES
- NO

1. I can take a rest break (to have a drink or meal).

Please select one answer.

- YES
- NO

Below is a list of statements related to your COVID-19 voluntary service. Please indicate how strongly you agree or disagree with them using a 5-point scale:

1. Volunteering allows me to learn new interesting things.

Please select one answer.

- strongly disagree
- disagree
- undecided
- agree
- strongly agree

1. Volunteering makes me feel needed.

Please select one answer.

- strongly disagree
- disagree
- undecided
- agree
- strongly agree

1. I believe that my skills may be of use to the community at this difficult time.

Please select one answer.

- strongly disagree
- disagree
- undecided
- agree
- strongly agree

1. Volunteering gives me an opportunity to meet new friends.

Please select one answer.

- strongly disagree
- disagree
- undecided
- agree
- strongly agree

1. I think it is important to help others.

Please select one answer.

- strongly disagree
- disagree
- undecided
- agree
- strongly agree

1. Volunteering is good for my future professional development.

Please select one answer.

- strongly disagree
- disagree
- undecided
- agree
- strongly agree

1. Volunteering increases my self-esteem.

Please select one answer.

- strongly disagree
- disagree
- undecided
- agree
- strongly agree

1. I have decided to volunteer mostly to receive a credit for internship.

Please select one answer.

- strongly disagree
- disagree
- undecided
- agree
- strongly agree

Please indicate the benefits of volunteering based on your experiences of COVID-19 voluntary service:

1. I am learning new medical skills.

Please select one answer.

- YES
- NO

1. I am learning new social skills.

Please select one answer.

- YES
- NO

1. I am learning new organisational skills.

Please select one answer.

- YES
- NO

1. I am learning new skills of dealing with stressful or difficult situations.

Please select one answer.

- YES
- NO

1. I am making new contacts.

Please select one answer.

- YES
- NO

1. I am receiving positive reactions from the people I help.

Please select one answer.

- YES
- NO

1. I am receiving positive reactions from the people I work with during my voluntary service.

Please select one answer.

- YES
- NO

1. I am receiving a positive response from the community / gaining social esteem.

Please select one answer.

- YES
- NO

1. I have found that I made the right choice of the field of study.

Please select one answer.

- YES
- NO

Submit 🡪
