# Supplementary material for: Student Volunteering as a Solution for Undergraduate Health Professions Education: Lessons From the COVID-19 Pandemic
Source: Front Public Health. 2021 Jan 26;8:633888. doi: 10.3389/fpubh.2020.633888 (PMC7871007; doi:10.3389/fpubh.2020.633888)
Supplement: Supplementary file 2 [file Table_2.DOCX]

Supplementary File 2. The COVID-19 student volunteering: questions for semi-structured interviews

1. **Benefits**: What were the benefits (or advantages) of participation in the student volunteering project at PUMS?
2. **Costs**: What were the costs (or disadvantages) of participation in the project?
3. **Safety and organisation**: What do you think of the safety and organisation of the project?
4. **Reasons for participation**: Why did you decide to participate in the project? In your opinion, why did other students participate?
5. **Opinion on the internship credit as an incentive**: What do you think of the decision to grant internship credits to project participants?
6. **Opinion on making student volunteering a curricular activity in the future**: What do you think of making student volunteering part of university curricula in the future?
